# Choices change the temporal weighting of decision evidence

**DOI:** 10.1152/jn.00462.2020

**Published:** 2021-03-10

**Authors:** Bharath Chandra Talluri, Anne E. Urai, Zohar Z. Bronfman, Noam Brezis, Konstantinos Tsetsos, Marius Usher, Tobias H. Donner

**Affiliations:** ^1^Department of Neurophysiology and Pathophysiology, University Medical Center Hamburg-Eppendorf, Hamburg, Germany; ^2^Department of Psychology, University of Amsterdam, Amsterdam, The Netherlands; ^3^School of Psychology, Tel-Aviv University, Tel-Aviv, Israel; ^4^Sagol School of Neuroscience, Tel-Aviv University, Tel-Aviv, Israel; ^5^Amsterdam Brain and Cognition Center, University of Amsterdam, Amsterdam, The Netherlands

**Keywords:** arousal, confirmation bias, decision-making, human, psychophysics

## Abstract

Many decisions result from the accumulation of decision-relevant information (evidence) over time. Even when maximizing decision accuracy requires weighting all the evidence equally, decision-makers often give stronger weight to evidence occurring early or late in the evidence stream. Here, we show changes in such temporal biases within participants as a function of intermittent judgments about parts of the evidence stream. Human participants performed a decision task that required a continuous estimation of the mean evidence at the end of the stream. The evidence was either perceptual (noisy random dot motion) or symbolic (variable sequences of numbers). Participants also reported a categorical judgment of the preceding evidence half-way through the stream in one condition or executed an evidence-independent motor response in another condition. The relative impact of early versus late evidence on the final estimation flipped between these two conditions. In particular, participants’ sensitivity to late evidence after the intermittent judgment, but not the simple motor response, was decreased. Both the intermittent response as well as the final estimation reports were accompanied by nonluminance-mediated increases of pupil diameter. These pupil dilations were bigger during intermittent judgments than simple motor responses and bigger during estimation when the late evidence was consistent than inconsistent with the initial judgment. In sum, decisions activate pupil-linked arousal systems and alter the temporal weighting of decision evidence. Our results are consistent with the idea that categorical choices in the face of uncertainty induce a change in the state of the neural circuits underlying decision-making.

**NEW & NOTEWORTHY** The psychology and neuroscience of decision-making have extensively studied the accumulation of decision-relevant information toward a categorical choice. Much fewer studies have assessed the impact of a choice on the processing of subsequent information. Here, we show that intermittent choices during a protracted stream of input reduce the sensitivity to subsequent decision information and transiently boost arousal. Choices might trigger a state change in the neural machinery for decision-making.

## Graphical abstract

**Figure:**
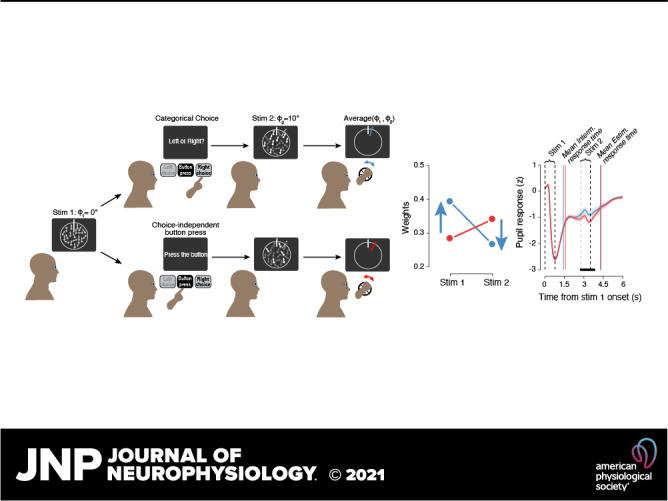

## INTRODUCTION

Many decisions need to be made on the basis of noisy, incomplete, or ambiguous decision-relevant information. An extensive body of research on perceptual decisions under uncertainty has converged on the idea that evidence about the state of the sensory environment is continuously accumulated across time (1, 2). In the choice tasks commonly used in the laboratory, performance is maximized by weighing evidence equally across time (1, but see 3–5). Yet, the evidence weighting applied by human and nonhuman decision-makers often deviates from such flat weighting profiles. Some studies found stronger weighting of early evidence (“primacy”; 6–9), others stronger weighting of late evidence (“recency”; 10–12), and yet others even nonmonotonic weighting profiles (13, 14). These distinct temporal weighting profiles may inform about differences in the mechanisms underlying decision formation (14–17). Yet, mechanistic inferences are limited by the fact that most of the above studies were conducted in different subjects and used various different stimuli and tasks (with evidence varying on different timescales). Thus, the resulting heterogenous weighting profiles may also reflect idiosyncratic strategies and/or task or stimulus differences. Demonstrations of changes in evidence weighting within subjects processing the same stimulus material are rare (15).

Real-life decisions are not isolated events but embedded in a sequence of judgments based on continuous streams of information, raising the question of whether and how successive decisions interact. Even in elementary perceptual decisions, postdecisional neural signals reflecting the previous choice have been identified in several regions of the monkey (18, 19) and rodent brain (20, 21). Likewise, perceptual choices are biased by the choices made on previous trials (22–37). Recently developed psychophysical protocols provide new tools for quantifying the effect of choices on the subsequent processing of decision evidence. These tasks prompt two successive judgments within the same trial: a binary choice, followed by a continuous estimation (38–43) or a confidence judgment (44, 45). In some of those tasks, the binary choice is followed by an additional evidence stream, enabling quantification of the impact of the choice on the processing of subsequent evidence (38, 42). These task designs have yielded two insights. First, the overall sensitivity to evidence following the intermittent choice is reduced in a nonselective fashion. Second, sensitivity for information consistent with the binary choice is selectively enhanced, at the expense of reduced sensitivity for choice-inconsistent evidence, yielding a bias to confirm the initial choice (confirmation bias; 42).

Here, we studied the relationship between the nonselective and selective changes in sensitivity following a choice and assessed their impact on temporal evidence weighting profiles. To this end, we reanalyzed the data sets from both these previous studies (38, 42). For one of the data sets, we also explored a relationship between these behavioral phenomena and pupil-linked, phasic arousal (35, 46–49).

## MATERIALS AND METHODS

### Behavioral Tasks

#### Perceptual task.

**Figure 1.**
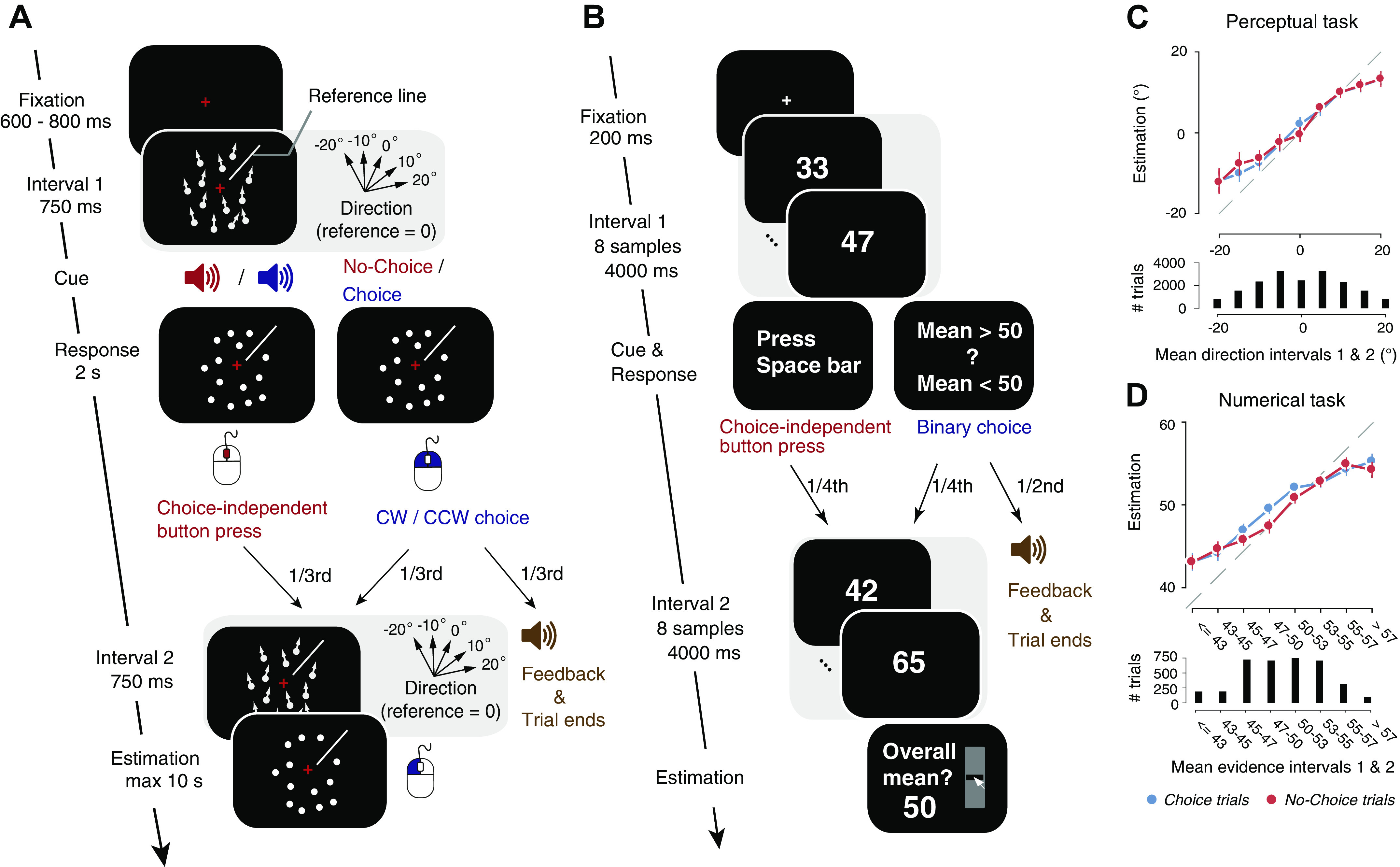
Behavioral tasks: schematic of sequence of events within a trial. *A*: Perceptual task. After a first random dot motion stimulus was shown for 750 ms, participants received an auditory cue about whether to report a binary choice about the net motion direction (Choice trials) or to continue the trial (No-Choice trials). The choice entailed discriminating the motion direction as clockwise (CW) or counterclockwise (CCW) with respect to the reference (white line shown at about 45° in this example). On half the Choice trials, auditory feedback was then given and the trial terminated. In the other half, and in all No-Choice trials, a second motion stimulus was presented (with equal coherence as the first but different direction), and participants were asked to report a continuous estimate of the mean direction of both stimuli by dragging a line along the screen with the mouse. Directions in the first and second intervals were sampled from one of the five possible values shown, with respect to the reference (0° corresponds to direction along the white reference line). See Supplemental Video S1 for a video of the task structure. *B*: Numerical task. After the first sequence of eight numerical samples, participants were instructed to either press the space bar (a quarter of all trials; No-Choice trials), or to give their binary choice about the average of the eight samples (mean >50 or <50; Choice trials). On two-thirds of Choice trials (constituting a half of all trials), auditory feedback was presented and the trial terminated. In the rest, a second sequence of eight numerical samples was presented and participants were instructed to give a continuous estimate of the mean across the two sequences. Adapted from Ref. 42 under a CC-BY license. *C*, *top*: continuous estimations as a function of mean direction across both stimuli in the perceptual task. *Bottom*: distribution of mean directions across trials. Data points, group mean; error bars, SE. Stimulus directions and estimations were always expressed as the angular distance from the reference, the position of which varied from trial to trial (0° equals reference). *D*: same as *C* but for Numerical task. Mean evidence across *intervals 1* and *2* in *D* split into discrete bins for illustration.

The University of Amsterdam ethics review board approved the study (reference number 2014-BC-3517). All participants gave their written informed consent. Participants were presented with two random dot motion stimuli in succession and were asked to estimate the average motion direction across the two intervals in each trial (Fig. 1*A*). A white line plotted on top of the circular aperture served as the reference, whose position changed between trials. An auditory cue after the first interval prompted the participants to make one of the two intermittent responses: *1*) report a binary choice about the direction of dots in the first interval (clockwise (CW) or counterclockwise (CCW) with respect to the reference; two-third proportion of all trials) by pressing left/right mouse buttons; or *2*) make a choice-independent button press (one-third proportion of all trials) by pressing the central mouse wheel. This intermittent response allowed us to investigate if participants showed different sensitivity to the second stimulus depending on whether they reported a binary choice (so-called “Choice trials”) or made a choice-independent motor response (so-called “No-Choice trials”). The position of the reference line for each participant was constrained to be within the top half or bottom half of the stimulus annulus (balanced across participants) to ensure a fixed choice (CW/CCW)–response (left/right buttons) mapping. The delay between the first and second stimuli was fixed (2 s), regardless of the reaction time of the subject. Participants gave their estimation response by dragging a red line around the circle using the mouse, starting from the reference line, and clicking the mouse at the end point. Half of all choice trials ended with an auditory feedback about the correctness of the binary choice to motivate participants to take the binary choice component seriously. An animation of the task structure is shown in the supplemental video file (Supplemental Video S1; all Supplemental material is available at https://doi.org/10.6084/m9.figshare.12752723). Feedback for the estimation response was provided at the end of each block (69 trials) as the mean deviation of the estimation report from the physical stimulus direction. The coherence of the stimuli was fixed at a predetermined level for each subject, and the direction of coherent dots in the two intervals was sampled independently from five possible values (−20°, −10°, 0°, 10°, 20° relative to the reference line). In all, 23 possible combinations of directions were used in the experiment (excluding the two most obviously conflicting directions: −20°/20° and 20°/−20°). A total of 90 trials for each combination of stimulus directions in the first and second intervals (45 Choice trials, and 45 No-Choice trials) were presented to the subjects. Subjects were not explicitly instructed about the distribution of stimuli. However, it is possible that subjects may have learned during the course of the task that the extremely inconsistent stimuli in the two intervals (+20 in *interval 1*, −20 in *interval 2* and vice versa) are unlikely in the task. However, such knowledge does not affect the sensitivity measures we computed since the stimulus sequences across both Choice and No-Choice conditions were counterbalanced for every subject (see Supplemental Fig. S1).

In all the analyses that follow, we used trials where participants made an estimation judgment (Choice trials and No-Choice trials). We excluded trials in which participants did not comply with the instructions, i.e., when they pressed the mouse wheel on Choice trials or a choice key on No-Choice trials, trials in which the binary choice response time was below 200 ms, and trials where estimations were outliers. Outliers were defined as those trials whose estimations fall beyond 1.5 times the interquartile range above the upper-quartile or below the lower-quartile of estimations. Together, these excluded trials corresponded to ∼7% of the total trials across participants. The distributions of trials used for analysis are shown in Supplemental Fig. S1, *A* and *B*.

We analyzed data from the same set of participants as in our earlier report (42). Please refer to this report for a detailed description of the task, participants, and stimuli used in the experiments.

#### Numerical task.

We reanalyzed data from the numerical integration task in Ref. 38 using the same analyses methods as the perceptual task. The task has a similar structure as the perceptual task (Fig. 1*B*), with the exception that participants saw 16 numerical samples displayed in succession and reported their mean as a continuous estimate. Like in the perceptual task, participants received a cue midway through the trial (i.e., after the first 8 samples) to either report a binary choice about the mean of the eight samples (greater or less than 50) or to make a choice-independent motor response (by pressing the space bar). In 50% of all trials, the trial terminated with feedback after the binary choice. On another 25% of the trials, participants saw the second stream of eight numerical samples and made the continuous estimation judgment at the end (Choice trials). In the rest 25% of trials, participants made a choice-independent motor response (No-Choice trials) instead of the binary choice and a continuous estimation judgment at the end. We analyzed data from all the trials where participants made the estimation judgment (50% of all trials).

The sequence of eight numbers in each interval was generated from one of the four predefined triangular skewed-density distributions with means of 40, 46, 54, or 60 (38). The numbers ranged between 10 and 90. Numbers were sampled such that two identical numbers were never presented in succession. The first eight numbers were sampled from one of the two distributions with means at 46 or 54, and the second eight numbers were independently sampled from one of the four distributions, randomized between trials. This ensured that subjects could not guess the mean of the second sequence of numbers after the first interval.

We analyzed data from 20 out of 21 participants participated in the study. The remaining subject (*subject 21*) failed to do the task (Spearman’s correlation between estimation and mean evidence in No-Choice trials: rho = 0.18, *P* = 0.117; and in Choice trials: rho = 0.17, *P* = 0.156). Please see the earlier reports (38, 42) for more detailed description of the task, stimuli, and participants.

### Pupillometry

Horizontal and vertical gaze position as well as pupil diameter were recorded at 1000 Hz using an EyeLink 1000 (SR Research). The eye tracker was calibrated before each block. Blinks detected by the EyeLink software were linearly interpolated from −150 ms to 150 ms around the detected velocity change. All further data analysis was done using FieldTrip (50) and custom Matlab scripts. We estimated the effect of blinks and saccades on the pupil response through deconvolution (35, 48, 51) and removed these responses from the data using linear regression. The residual pupil signal was bandpass filtered from 0.01 to 10 Hz using a second-order Butterworth filter, *Z*-scored per block of trials, and downsampled to 50 Hz. We extracted epochs of the pupil time series comprising the trials of the behavioral task and baseline corrected the pupil time-course in each trial by subtracting the mean pupil diameter 500 ms before the onset of the first evidence stream on each trial. This yielded a single measure of the task-evoked pupil response as used in previous studies (35, 48).

Within each experimental condition (Choice, No-Choice, Consistent, Inconsistent), we averaged the corrected pupil responses across trials, time-locked to the onset of the first evidence sequence and spanning up to the onset of the next trial, or a maximum duration of 6 s.

### Modeling Discrimination Judgments

Participants’ binary choices in the perceptual and numerical tasks were modeled using a sigmoidal probit psychometric function, relating the proportion of CW choices (>50 choices in the numerical task) to the stimulus direction (mean of the 8 numerical samples) in the first interval, as follows:

(*1*) $$P\left(Choice=CW|\;\right|\phi_{1})=\;\Phi (\delta +\;\alpha \phi_{1})$$

where *ϕ*_1_ was the stimulus direction (mean of 8 numerical samples in Numerical task) in the first interval, $\Phi \left(x\right)=\;\frac{1}{\sqrt{2\pi }}\overset{x}{\underset{-\infty }{\int }}e^{-t^{2}/2}dt$ was the cumulative Gaussian function, *α* was the slope of the psychometric function (perceptual sensitivity), and *δ* was the horizontal shift of the psychometric function (systematic bias toward one of the two choice alternatives). The free parameters *α* and *δ* were estimated using maximum likelihood estimation (52). In the data from the numerical task, the sample means from the first interval were binned into six bins, three on each side of the reference (50) before fitting the psychometric function.

### Modeling Estimation Reports

#### General approach.

We used a statistical modeling approach to estimate the relative contribution of the evidence (i.e., physical stimulus or numerical evidence corrupted by noise) conveyed by successive dot motion stimuli in each interval (mean of numerical samples in each interval in the Numerical task) to participants’ trial-by-trial estimation reports. The noisy sensory (or numerical) evidence was described by:

(*2*) $$X_{i}=\phi_{i}+\delta +N\left(0,\sigma^{2}\right)\;$$

where *i* ∈ (1, 2) denotes the interval, *ϕ_i_* is the physical stimulus direction (or the mean of 8 numerical samples in the Numerical task), *N* (0, *σ*^2^) was zero mean Gaussian noise with standard deviation *σ* (= 1/*α*), *δ* and *α* were each observer’s individual overall bias and sensitivity parameters respectively taken from the psychometric function fit to the binary choice data (*Eq. 1*).

### Global Gain Model

We modeled a global, choice-related change in sensitivity to evidence following an overt choice, by allowing the weights to vary separately in Choice trials and No-Choice trials. The estimations were modeled by:

(*3.1*) $$y_{c}=w_{1c}X_{1c}+w_{2c}X_{2c}+N\left(0,{\xi_{c}}^{2}\right)$$

(*3.2*) $$y_{nc}=w_{1nc}X_{1nc}+w_{2nc}X_{2nc}+N\left(0,{\xi_{nc}}^{2}\right)$$

where ***y_c_*** (***y_nc_***) was the vector of estimations across all Choice (No-Choice) trials, *w*_1_*_c_* (*w*_1_*_nc_*) and *w*_2_*_c_* (*w*_2_*_nc_*) were the weights for the noisy evidence ***X***_1_***_c_*** (***X***_1_***_nc_***) and ***X***_2_***_c_*** (***X***_2_***_nc_***) encoded in *intervals 1* and *2* in Choice (No-Choice) trials, respectively. *N* (0, *ξ*^2^) was zero-mean Gaussian estimation noise with variance *ξ*^2^ that captured additional noise in the estimations, separately for Choice (*ξ_c_*), and No-Choice (*ξ_nc_*) trials, over and above the sensory noise corrupting the binary choice.

### Quantifying Confirmation Bias in Choice Trials

Using data from the sensory and numerical tasks, we showed in our previous work that people exhibited confirmation bias by overweighting evidence in the second interval that was consistent with the intermittent binary choice (42). To this end, we fitted a model referred to as “choice-based selective gain” model to Choice trials (42). The model is described below:

(*4.1*) $$y_{cc}=w_{1cc}X_{1cc}+w_{2cc}X_{2cc}+N\left(0,{\xi_{cc}}^{2}\right)$$

$$if\;\;\;\;sign\left(\phi_{2}\right)=\;D\;$$

(*4.2*) $$y_{ic}=w_{1ic}X_{1ic}+w_{2ic}X_{2ic}+N\left(0,{\xi_{ic}}^{2}\right)$$

$$if\;\;\;\;sign\left(\phi_{2}\right)\neq \;D\;$$

where *w*_1_*_cc_* (*w*_2_*_cc_*) and *w*_1_*_ic_* (*w*_2_*_ic_*) were the weights, *ξ_cc_* and *ξ_ic_* were the estimation noise parameters for Consistent and Inconsistent trials, respectively, **ϕ_2_** was the vector of physical stimulus directions (mean of 8 samples in the Numerical task) from the second interval, and **D** was the vector of intermediate binary choice (values: 1 or −1 for CCW and CW reports in the perceptual task, and for <50 and >50 reports in the Numerical task, respectively). We excluded the subset of trials where consistency is not defined in *Eq. 4*, i.e., *ϕ*_2_ = 0° in Perceptual task, and 50 in the Numerical task. Confirmation bias is quantified as the difference in the weights *w*_2_*_cc_* and *w*_2_*_ic_*.

### Fitting Procedure

The models described in *Eqs. 3* and*4* assume that the stimuli and estimation judgments are corrupted by Gaussian noise. Thus, on a given trial (comprising a unique combination of experimental variables: first and second stimuli and choice), the estimation that is predicted by a certain model parametrization is a continuous random variable characterized by a probability density function (hereafter, the estimation distribution). Similar to our earlier report (42), we fitted the models to the data using a maximum likelihood approach. For each trial, we calculated the log-likelihood of the estimation judgment provided by the participant given the estimation distribution predicted by the model. Using the Subplex algorithm (53, 54), which is a generalization of the Nelder–Mead simplex method, we searched for the parameter set that maximized the sum of the log-likelihood values across all trials.

Importantly, we obtained the estimation distributions of the models numerically, which allowed us to estimate maximum likelihood parameters accurately without relying on stochastic stimulations. To illustrate our numerical approach, we next describe how the estimation distribution is calculated in the global gain model (*Eqs. 3.1* and *3.2*). In a No-Choice trial, the estimation predicted by the model is the sum of three Gaussian variables (*Eq. 3.2*): the weighted noisy (see *Eq. 2*) representation of the stimulus in *interval 1* (*w*_1*nc*_***X***_1***nc***_∼***N***(*w*_1*nc*_(***ϕ***_1_ + ***δ***), *w*_1*nc*_^2^σ^2^)), the weighted noisy representation of the stimulus in *interval 2* (*w*_2*nc*_***X***_2***nc***_∼***N***(*w*_2*nc*_(***ϕ***_2_ + ***δ***), *w*_2*nc*_^2^σ^2^)), and estimation noise (*N*(0, ξ_*nc*_^2^)). The estimation report, being the sum of three Gaussian variables, is a Gaussian variable itself with the following density function (or estimation distribution):

$$Y_{nc}∼N\left(w_{1nc}{(\varphi }_{1}+\delta \right)+w_{2nc}{(\varphi }_{2}+\delta ),\sigma^{2}({w_{1nc}}^{2}+\;{w_{2nc}}^{2})+{\xi_{nc}}^{2}\text{).}$$

Akin to a No-Choice trial, in a Choice trial, the estimation report is also the sum of three random variables (*Eq. 3.1*). In contrast to a No-Choice trial, however, the weighted noisy representation of the stimulus in *interval 1* is conditioned upon the categorical choice that was given at the end of that interval. Specifically, conditioning upon the choice means that the density of the ***N***(*w*_1*c*_(***ϕ***_1_ + ***δ***), *w*_1*c*_^2^σ^2^) probability function is set to zero for all positive (negative) stimulus values, if a negative (positive) categorical choice was made (positive refers to the side on the right of the reference and negative to the side on the left). This density truncation captures the fact that the noisy instantiation of the *interval 1* stimulus gives rise (and thus cannot be incongruent) to the categorical choice. Due to the truncation, the weighted noisy representation of the *interval 1* stimulus is a non-Gaussian variable and, accordingly, the estimation report in Choice trials is also a non-Gaussian variable. To derive the estimation distribution, we thus numerically convolved the *interval 1* non-Gaussian density function with the other two Gaussian density functions [*w*_2*c*_***X***_2***c***_∼***N***(*w*_2*c*_(***ϕ***_2_ + ***δ***), *w*_2*c*_^2^σ^2^) and *N*(0, ξ_*c*_^2^)]. A similar convolution approach was used when deriving the estimation distribution of the selective gain model (*Eq. 4*).

### ROC Analysis for Differences in Sensitivity to Evidence in Interval 2

We assessed the impact of an overt choice on the evidence in *interval 2* from participants’ estimations in a model-free fashion, using the so-called ROC analysis. This analysis was based on the receiver operating characteristic (ROC) (55), similar to the one used in our earlier report (see “model-free analysis of estimation reports” in 42). The ROC index quantified the extent of separability between two distributions, with the value 0.5 if the distributions were identical, and 1 if they were completely separable. By computing ROC indices between estimation distributions of sets of trials that differed in their input, we could assess the sensitivity of the observer in using that input to guide their estimation reports.

For the perceptual task, in each condition (Choice and No-Choice), we first sorted trials based on the directional evidence in *interval 1* (*ϕ*_1_). For each *ϕ*_1_, we ran the ROC analysis on all pairs of estimation distributions, separated by 10° of directional evidence in *interval 2* (*ϕ*_2_): −20° versus −10°, −10° versus 0°, 0° versus 10°, and 10° versus 20°. This gave us four ROC indices per *ϕ*_1_, one index for every pair of distributions compared. We then computed a weighted average ROC index for each *ϕ*_1_, weighting the individual ROC indices by the number of trials that went into the ROC analysis. This approach ensured that the resulting ROC indices are robust to changes in *ϕ*_1_. These indices are averaged to obtain one single ROC index per subject for each condition.

ROC indices for the numerical task were computed similar to the above procedure with the following exceptions: mean evidence in *interval 1* and *interval 2* were binarized (mean >50 or mean <50) resulting in two bins for *interval 1* and *interval 2*, respectively. We obtained qualitatively similar results using four bins to compute ROC indices. These binarized values were treated equivalent to *ϕ*_1_ and *ϕ*_2_ in the perceptual task above.

### Simulated Estimation from Best Fitting Parameters

We simulated estimations for Choice trials using the trial distributions and best fitting parameters of the Global Gain model (*Eq. 3.1*) in individual participants in both the tasks. To this end, we first calculated the noisy representations *X*_1_ and *X*_2_ corresponding to *intervals 1* and *2*, using *Eq. 2* above. We ensured that the sign of representations in *X*_1_ matches the binary choices of subjects (by randomly sampling the representations until a sample with the correct sign as that of the subjects’ choice was obtained), and then combined the representations from both intervals with the corresponding parameters for each individual subject using *Eq. 3*.*1*. The choice-based selective gain model (*Eqs. 4*.*1* and *4*.*2*) was fit to the simulated estimations to recover consistent and inconsistent weights (Fig. 4*B*).

### Confidence Intervals for Individual Measures

We used bootstrapping (56) to obtain confidence intervals for the fitted parameters and ROC indices for each subject. Within each subject, we randomly selected trials with replacement and computed the model-based and model-free metrics on the bootstrapped data set. To compute model weights, we used the same fitting procedure but using the best fitting parameters of the actual data as starting points. We repeated this procedure 500 times and obtained confidence intervals from the distribution of estimated metrics.

### Computation of Correlation Values

We used Pearson’s correlation coefficients to examine how different measures are related to each other in each task. We then estimated the confidence intervals of the correlation coefficients by generating a distribution of 10,000 bootstrapped Pearson’s correlations for each task. To compute each of these correlations, we randomly sampled, with replacement, the pair of parameters to be correlated from the bootstrapped estimates in every subject. We repeated this process 10,000 times and computed the confidence intervals from this distribution.

To obtain the correlation values for data pooled from both of the tasks (Figs. 3 and 4), we first obtained Pearson’s correlation coefficient for data set from each task (also reported in the figures). We then applied Fisher transformation on the correlation values and calculated their weighted average to obtain the pooled Fisher-transformed correlation coefficient. This quantity was used to obtain the pooled Pearson’s correlation coefficient (using inverse Fisher transformation). To obtain confidence intervals for the pooled correlation coefficient, we generated a distribution of 10,000 bootstrapped pooled correlation coefficients by combining 10,000 randomly sampled (with replacement) bootstrapped correlation coefficients in the Perceptual task data set with 10,000 randomly sampled (with replacement) bootstrapped correlation coefficients in the Numerical data set. We computed the confidence intervals of the pooled correlation coefficient from this distribution.

To compare the difference between two correlation coefficients (Fig. 5, *C* and *D*), we applied Fisher-transformation on the correlation coefficients and computed the absolute difference between the Fisher-transformed correlation coefficients. This quantity was used to obtain the corresponding *P* value.

### Statistical Tests

Nonparametric permutation tests (56) were used to test for group-level significance of individual measures for each task, unless otherwise specified. This was done by randomly switching the condition labels of individual observations between the two paired sets of values in each permutation. After repeating this procedure 100,000 times, we computed the difference between the two group means on each permutation and obtained the *P* value as the fraction of permutations that exceeded the observed difference between the means. All *P* values reported were computed using two-sided tests, unless otherwise specified.

For the Perceptual task, the number of unique permutations was 2^10^ = 1,024, substantially less than the above number of permutations. All qualitative results reported here were unchanged when using exactly these 1,024 unique permutations of condition labels to compute the permutation distribution as described above.

We used repeated measures two-way analysis of variance (ANOVA) with interval and condition (Choice, No-Choice) as factors to test for group-level significance of the interaction between condition and interval for both the tasks (Fig. 3*A*). We used standard parametric methods to assess statistical significance of correlation coefficients.

### Data and Analysis Code

Behavioral data for the Perceptual Task are available at https://doi.org/10.6084/m9.figshare.7048430 (42) and for the Numerical Task are available at https://datadryad.org/resource/doi:10.5061/dryad.40f6v (38). Raw pupil data from the Perceptual task are made available at https://doi.org/10.6084/m9.figshare.14039294. Analysis code reproducing all the analyses in the paper is made available at https://github.com/BharathTalluri/choice-commitment-bias.

## RESULTS

Participants reported a continuous estimate of the mean of fluctuating sensory (perceptual task, Fig. 1*A*, Supplemental Video S1) or symbolic (numerical task, Fig. 1*B*) evidence across two successive intervals. This estimate needed to be based on integrating some internal representation of the fluctuating evidence—motion direction or numerical value in the perceptual or numerical tasks, respectively—across the two stimulus intervals.

On a subset of trials (so-called Choice trials), participants were also asked to report an intermediate choice after the first stimulus: a fine direction discrimination judgment relative to a visually presented reference line (Perceptual Task) or comparison of the numerical mean with 50 (Numerical Task). On the remaining set of trials (No-Choice trials), participants were asked to press a button for continuing the trial, without judging the first evidence stream. The cue informing participants whether to report the discrimination judgment or to press a choice-independent button press came after the first stimulus interval. This design enabled us to quantify the degree to which evidence in each interval contributed to the final estimation and whether this depended on the overt report of a categorical choice (see materials and methods).

The mean accuracy of the intermediate choice was 81% ± 10.8% (mean ± SD) for the perceptual task and 67% ± 6.7% for the numerical task. Estimation responses in both tasks increased with mean directional evidence across the two intervals (Fig. 1, *C* and *D*) and did not differ between Choice and No-Choice trials, with negligible and statistically nonsignificant differences in the regression slopes for estimations as a function of mean evidence (perceptual task: 0.0256, *P* = 0.8449; numerical task: 0.0125, *P* = 0.9083).

### Down-Weighting of Evidence following Intermittent Choice

**Figure 2.**
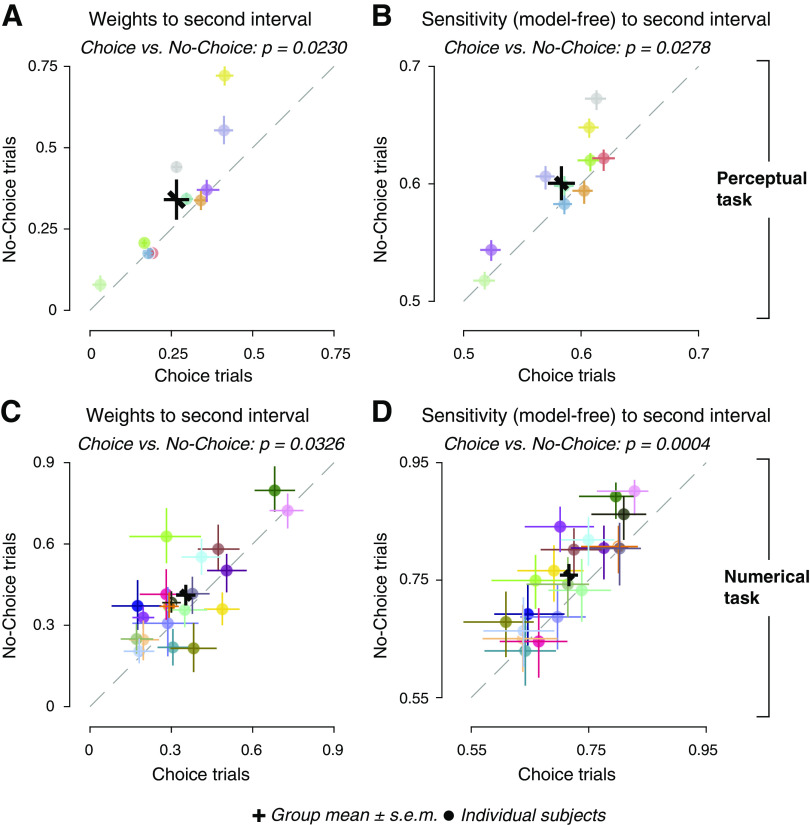
Sensitivity to second stimuli in Choice and No-Choice trials. *A*: model weights for sensitivity to second interval in Choice and No-Choice conditions in Perceptual task. Black cross, mean and SE; data points, individual participants, with identical color scheme from *A* and *B*; error bars on each data point, 66% bootstrap confidence intervals; dashed line, identity of Choice and No-Choice; points above diagonal indicate larger weights to No-Choice. *B*: same as *C*, but for receiver operating characteristic (ROC) indices quantifying the sensitivity to second interval in a model-free way in Perceptual task. *C* and *D*: same as *A* and *B*, but for Numerical task. Perceptual task, *n* = 10 participants; Numerical task, *n* = 20 participants; *P* values, permutation tests across participants (100,000 permutations).

We previously found lower sensitivity to subsequent evidence in the Choice condition compared with the No-choice condition in the Numerical Task (38). Here, we replicated this pattern of results, now also for the Perceptual Task, using a somewhat different statistical modeling approach as well as a model-free approach based on ROC analysis (see materials and methods). Both approaches quantified the sensitivity of the final estimation judgments for evidence in each interval. Across participants, model weights for the second evidence stream were significantly smaller in Choice trials compared with No-Choice trials (Fig. 2, *A* and *C*). Likewise, a model-free measure of sensitivity to subsequent evidence (area under the ROC curve) was smaller on Choice trials compared with No-choice trials (Fig. 2, *B* and *D*). This shows that the reduction in sensitivity following a categorical judgment generalizes from the domain of numerical to perceptual decision-making.

### Flip of Temporal Weighting of Sensory Evidence between Choice and No-Choice Conditions

**Figure 3.**
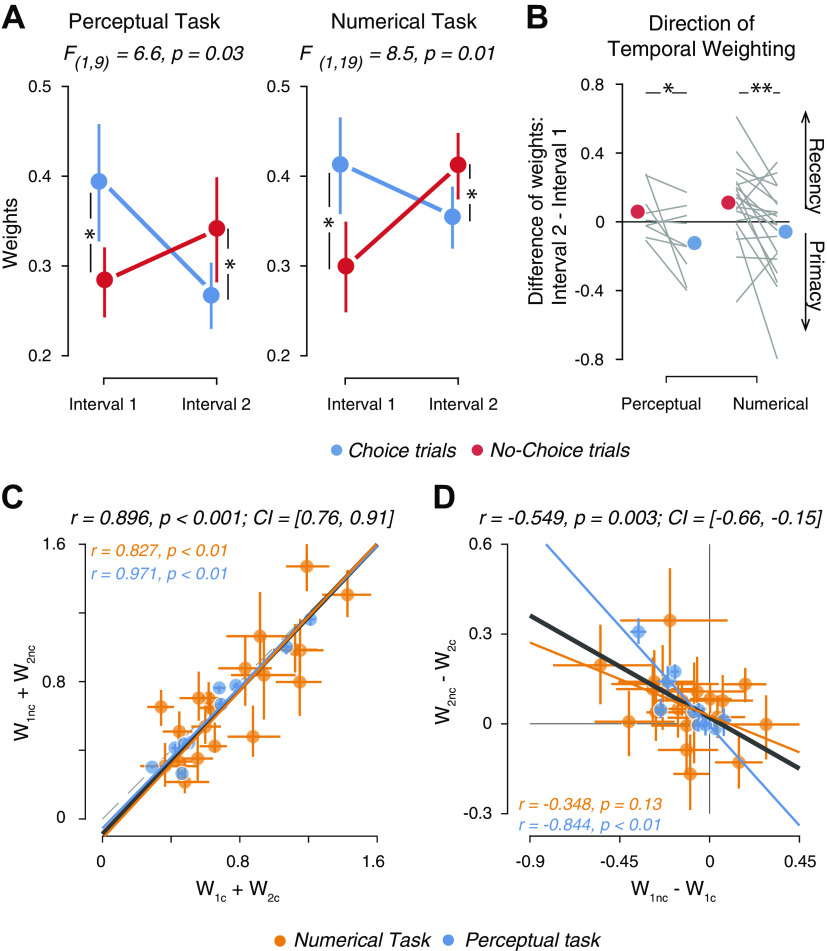
Choice-dependent alteration of temporal weighting profiles. *A*: mean model weights for both stimulus intervals in Choice and No-Choice conditions in Perceptual task (*left*, *n* = 10 participants) and Numerical task (*right*, *n* = 20 participants). Error bars, SE; *F*-statistic, interaction between interval and condition (repeated measures 2-way ANOVA). *B*: direction of temporal weighting quantified as difference in model weights between *interval 2* and *interval 1*, separately for each task; *P < 0.05, **P < 0.01, permutation tests across participants (100,000 permutations). *C*: sum of weights across both intervals in Choice and No-Choice conditions, across participants from both tasks. *D*: difference between weights in Choice condition and No-Choice condition, in both intervals across participants from both tasks. Data points, individual participants; error bars on each data point, 66% bootstrap confidence intervals; solid lines, best fitting lines; *r*, Pearson’s correlation coefficients along with 95% bootstrap confidence intervals. CI, confidence interval; *w*_1_*_c_*, weight for the noisy evidence in *interval 1* in Choice trial; *w*_1_*_nc_*, weight for the noisy evidence in *interval 1* in No-Choice trial; *w*_2_*_c_*, weight for the noisy evidence in *interval 2* in Choice trial; *w*_2_*_nc_*, weight for the noisy evidence in *interval 2* in No-Choice trial.

We next assessed if and how the intermittent choice affected the relative weighting of early versus late evidence in the decision process underlying the final estimation judgments. For both tasks, the weights in Choice trials were higher than in No-Choice trials for the first interval, and lower than in No-Choice trials for the second interval (Fig. 3*A*). Correspondingly, we found a significant interaction between trial type (Choice vs. No-Choice) and interval for both tasks (Fig. 3*A*, see Supplemental Fig. S2 for the corresponding interaction in ROC indices). This effect could also explain the similarity in overall estimation accuracy between Choice and No-Choice conditions (Fig. 1, *C* and *D*).

The interaction yielded a marked change in the temporal evidence weighting profiles across both intervals: a flip from recency in the No-Choice condition to primacy in the Choice condition (Fig. 3*B*). This flip was evident in the individual data: while the sums of weights from both intervals were highly similar for Choice and No-Choice trials in each subject (Fig. 3*C*), the *difference* in Choice and No-Choice weights was negatively correlated between intervals (Fig. 3*D*). No such constraint was imposed in the statistical models used to estimate the weights (see materials and methods). These results indicate the distribution of a limited cognitive resource across both intervals: The intermittent choice after the initial evidence boosted sensitivity to that early evidence, at the cost of reducing sensitivity to subsequent evidence.

### Non-selective Sensitivity Reduction Is Coupled to Selective Confirmation Bias

**Figure 4.**
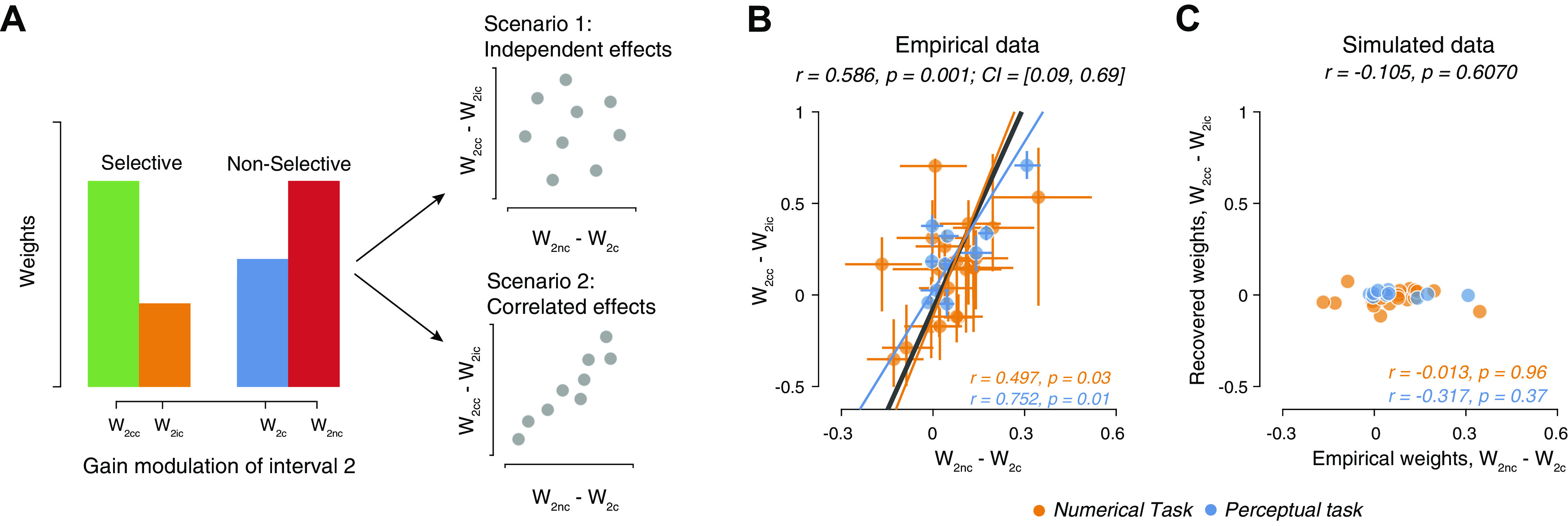
Relationship between global and consistency-dependent sensitivity modulation of subsequent evidence. *A*: schematic of two alternative relationships between the magnitudes of the observed nonselective and selective sensitivity modulations. *Scenario 1*: the two effects are produced by distinct mechanisms and thus their magnitudes are independent across subjects. *Scenario 2*: the two effects result from a common underlying mechanism and are therefore correlated across subjects. *B*: relationship between nonselective (quantified as the difference in weights to second interval between No-Choice and Choice conditions) and selective sensitivity modulation giving rise to Confirmation bias [quantified as the difference in weights to second interval between Consistent and Inconsistent conditions (42)], across participants from both tasks. *C*: same as *B*, but for simulated data. The parameters of the selective gain model (Consistent and Inconsistent weights) were estimated from data simulated using the best-fitting parameters of the Global Gain model for each participant (see materials and methods). Data points, individual participants; error bars on each data point, 66% bootstrap confidence intervals; solid lines, best fitting lines; dashed lines, identity lines; *r*, Pearson’s correlation coefficients; CI, 95% bootstrap confidence intervals for the correlation coefficients. *w*_2_*_c_*, weight for the noisy evidence in *interval 2* in Choice trial; *w*_2_*_cc_*, weight for the noisy evidence in *interval 2* in Consistent trial; *w*_2_*_ic_*, weight for the noisy evidence in *interval 2* in Inconsistent trial; *w*_2_*_nc_*, weight for the noisy evidence in *interval 2* in No-Choice trial.

Our previous analyses of the Choice conditions in the same data sets have shown that sensitivity is selectively enhanced for information consistent with the intermittent choice and reduced for choice-inconsistent evidence, yielding a bias to confirm the initial choice (42). We wondered whether the individual degree of this selective gain modulation (confirmation bias) was related to the nonselective gain modulation (overall reduction in sensitivity) applied to new evidence. We quantified the overall gain modulation as the difference in weights of *interval 2* between the Choice trials and the No-Choice trials, and the selective gain modulation as the difference in weights of *interval 2* between trials with choice-consistent and choice-inconsistent evidence (Fig. 4). These two gain modulations could be independent, suggesting different mechanistic bases for the modulations, or tightly correlated across subjects, suggesting a common underlying mechanism (Fig. 4*A*). We found the latter to be the case in the data: participants with a stronger global gain reduction also showed a stronger selective gain modulation (Fig. 4*B*). This was neither trivial (both are conceptually distinct effects) nor was it an artefact of our fitting procedure. We ruled out fitting artefacts by recovering the selective gain weights from data produced by simulating the Global gain model. In stark contrast to the empirical data (Fig. 4*B*), the recovered (from Global gain model simulations) selective gain effect was uncorrelated with the empirical nonselective gain effect (Fig. 4*C*).

### Pupil Responses during Decisions Reflect Cognitive Factors Shaping Evidence Reweighting

Taken together, the behavioral effects are in line with a change in the state of the decision-making machinery brought about by the initial choice. Such state changes may emerge from the recurrent dynamics of cortical decision circuits alone (57, 58). Another factor that may instigate a state change is the transient neuromodulatory input from central arousal (e.g., locus coeruleus noradrenaline) systems that occurs during decisions (48, 59–61). Such a neuromodulatory transient may be useful when the choice is prompted before decision circuits have reached a stable decision state through evidence accumulation (1)—a condition likely to hold for the intermittent choice in our task.

**Figure 5.**
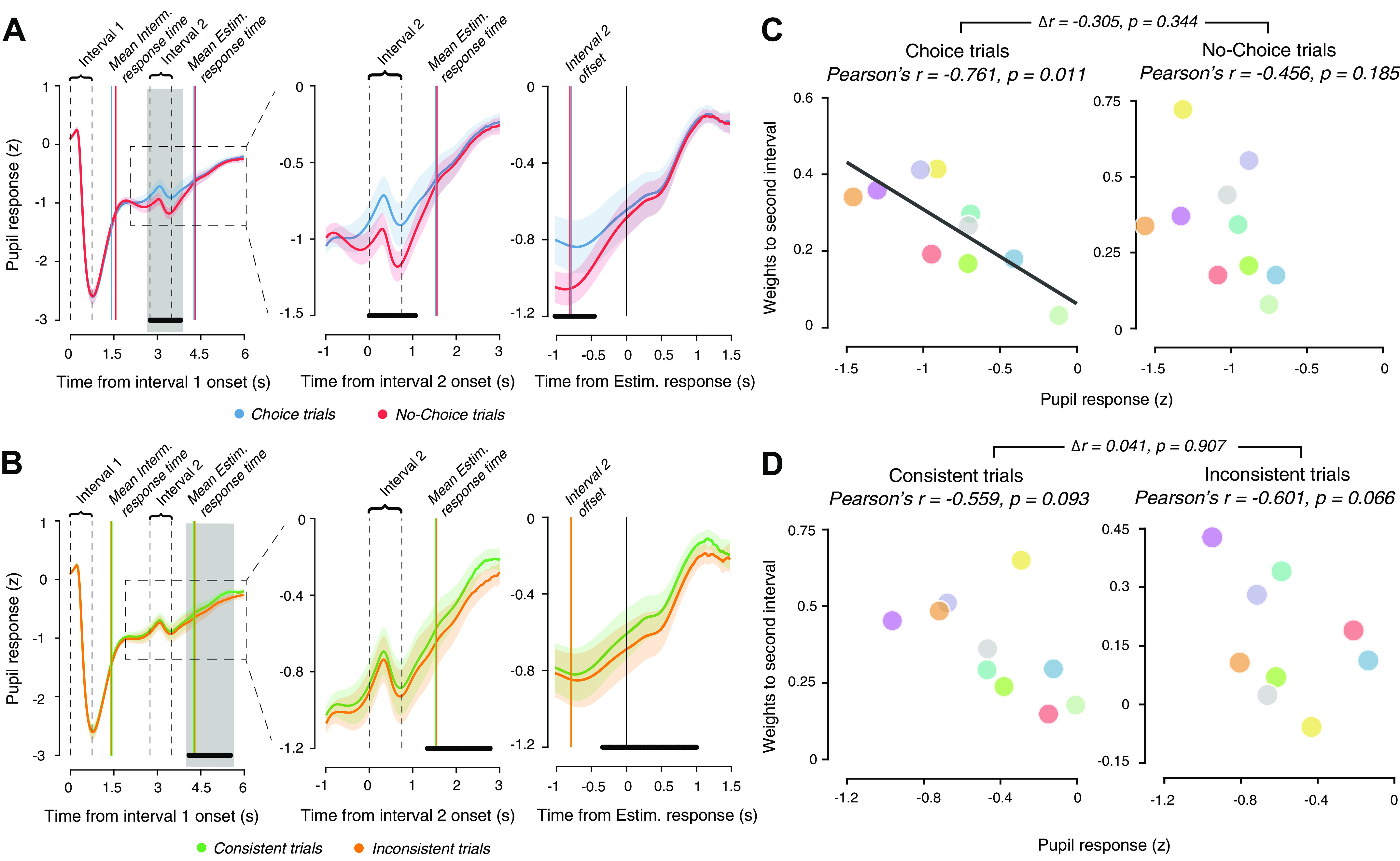
Evoked pupil responses reflect cognitive factors that drive evidence reweighting. *A*: time courses of average pupil diameter aligned to onset of *interval 1* for Choice and No-Choice conditions in the Perceptual task. *Left*: average time course across whole trial. *Middle*: close-up of time course during second stimulus interval (following intermediate response). *Right*: time-course aligned to estimation response (following second stimulus interval). Solid vertical lines after *interval 1*, mean intermittent response times across participants; solid vertical lines after *interval 2*, mean estimation response times across participants; dashed gray vertical lines, different events during the trial. All panels: solid lines, mean across participants; shaded region, SE; black horizontal bars, *P* < 0.05, cluster-based permutation test Choice vs. No-Choice. *B*: same as *A*, but for Consistent and Inconsistent trials. *C*: relationship between pupil response (computed as the mean pupil response in the gray-shaded time window in *A*) and model weights to second interval, in Choice trials (*left*) and in No-Choice trials (*right*). Solid line, best fitting line. *D*: as *C*, but for pupil responses and model weights in Consistent or Inconsistent trials and for the gray interval from *B*.

These considerations led us to analyze participants’ pupil size during the perceptual task (no pupil data were recorded in the numerical task). Nonluminance-mediated dilations of the pupil are a marker of the responses of brainstem arousal systems (48, 59, 62–64). As expected, the random dot stimulus at the start of trial elicited a pupil constriction, which was invariant between Choice and No-Choice trials (Fig. 5*A*, *left*, “*interval 1*”), reflecting the pupil response to retinal illumination (65). The initial pupil constriction was followed by a smaller increase in pupil diameter during the second interval following the intermittent response, and a larger response following the estimation report at the end of trial (Fig. 5, *A* and *B*). These later, nonluminance-mediated pupil responses may be due to cognitive factors and/or the motor responses associated with the behavioral reports of both decisions (47, 48, 66).

Critically, the pupil response after the intermittent response was bigger for Choice than No-Choice trials (Fig. 5*A*, *middle*), an effect that can neither be explained by the motor responses which occurred in both conditions, nor by the longer response times in the Choice condition [and the associated longer accumulation of central inputs in the peripheral pupil apparatus (47, 48)]: response times were, in fact, shorter in Choice than No-choice trials (see blue and red vertical lines respectively in Fig. 5*A*; permutation test, *P* = 0.0112). Thus, the stronger pupil response during Choice than No-Choice trials likely reflected the intermittent decision (47–49).

Pupil responses after *interval 2* exhibited another cognitive effect, being larger for Consistent trials than Inconsistent trials emerging ∼500 ms before the estimation reports (Fig. 5*B*, *right*). Due to the delay of the pupil response relative to the central arousal response (47–49), the differential arousal response likely emerged during the second interval of evidence processing, here dependent on the consistency of the evidence with the preceding choice. Again, this difference could not be explained by response times for the estimation reports, which were about equally long in both conditions (group mean response times: 0.78 s and 0.79 s for Consistent and Inconsistent trials respectively; condition difference: *P* = 0.4532, permutation test). In sum, pupil responses in different phases of the trial reflected the cognitive factors that were associated with the dynamic changes in evidence weighting: the need to report an initial judgment of the first evidence stream (Fig. 5*A*) and the consistence of the second evidence stream with the initial judgment (Fig. 5*B*).

### Association between Evoked Pupil Responses and Evidence Sensitivity

We finally performed exploratory analyses relating the pupil responses in the respective two trial intervals to the evidence weighting as inferred from our behavioral analysis. The complex and prolonged temporal profile of evoked pupil responses in our task complicates the within-subject analyses of this association at the single-trial level, which have proved useful in the context of simpler tasks with more transient responses (48, 49, 67). Specifically, the ramping of the pupil during the final estimation judgment was protracted well into the baseline interval of subsequent trials (Fig. 5, *A* and *B*), and thus affected single-trial baseline measurements in a fashion that complicates the quantification of single-trial response amplitudes. In rough first analyses, we found no systematic within-subject associations between evidence sensitivity for the second interval and the amplitude of pupil responses (data not shown) but did not pursue this further due to these complexities.

Across-subjects correlations of the mean pupil responses and model weights for the different conditions were not affected by this problem but were limited by the comparably low number of individuals measured in the perceptual task. We found a strong, negative across-subjects correlation between mean pupil response following the intermittent report and the evidence from the second interval in the Choice condition (Fig. 5*C*, *left*), but not the No-Choice condition (Fig. 5*C*, *right*). The difference in pupil response amplitude between Choice and No-Choice conditions did not predict the corresponding difference between second-interval weights in these two conditions (Pearson’s *r* = −0.205, *P* = 0.5697). We did not find an across-subjects correlation between mean pupil response amplitudes during the second interval on Consistent or Inconsistent trials and the corresponding weights for the second evidence (Fig. 5*D*).

## DISCUSSION

Recent work has begun to expose the impact of choices on the accumulation of subsequent decision evidence, revealing an overall reduction in sensitivity to subsequent evidence (38) combined with a selective suppression of the gain of evidence inconsistent with the initial choice (confirmation bias; 42). Here, we extended this nascent line of work, by showing that an intermittent discrimination judgment about an evidence stream introduces a change in the temporal weighting of the evidence on a final estimation judgment from recency to primacy, compared with an intermittent behavioral response independent of the evidence. We also showed that the above three effects are tightly related, consistent with a common underlying mechanism. Finally, we have found that these behavioral phenomena are accompanied by pupil-linked arousal responses that are modulated by the same factors that produce the evidence reweighting: intermittent choice and consistency of later evidence with that choice.

The here-discovered, strong relationship between the individual strength of the choice-induced, global sensitivity reduction and choice-induced, selective confirmation bias is not a given. Both effects were operationalized in terms of two orthogonal comparisons: the choice-induced sensitivity reduction by comparing sensitivity between trials with an intermittent choice and trials without such a choice; the confirmation bias by comparing trials with subsequent evidence that was consistent or inconsistent with the choice, *within* the trials that contain an intermittent choice. Thus, the presence of a global sensitivity reduction effect does not imply presence of the confirmation bias, and vice versa. Even so, their correlations were tight, in line with a common underlying mechanism.

The present data add to a growing body of literature indicating that the dynamics of evidence weighting for decision-making is highly context dependent. Both the timescale and the temporal profile of this weighting applied to the same physical evidence are affected by a range of factors including the type of judgment (68, 69), the type of information available in the stimulus (15), reliability of the sensory information (70), and the temporal statistics of the environment (3, 5, 71). We here extend this body of evidence, by showing that the temporal weighting profile, within a given individual and a given task, can be flipped by asking the participant for an intermittent choice in the middle of the evidence stream. Previous studies characterizing the dynamics of evidence accumulation during decision-making reported diverse temporal weighting profiles ranging from recency to uniform to primacy. But the differences between these studies in the stimuli, task protocols, and participants have complicated direct comparisons and mechanistic conclusions. Along with other recent findings (15), our findings establish that these temporal weighting profiles are neither fixed task properties nor fixed traits of decision-makers and lend themselves to mechanistic interpretation.

One possibility is that the effects of intermittent choices identified here are behavioral signatures of decision-related cortical circuit dynamics (57, 58, 72). Previous studies showed that these dynamics were task dependent and exhibit distinct state trajectories depending on whether the judgment is coarse categorization or fine discrimination (73). In a protracted task involving sequential categorical and estimation judgments such as ours, it is possible that the same decision circuits underlie both judgments. Once these decision circuits have settled in an attractor (choice commitment), this will reduce the network’s sensitivity to all new evidence (1, 57, 58, 74)—an effect that may hold regardless of whether that evidence is consistent or inconsistent with the choice (also see Ref. 38, Supplement). In this scenario, the attractor also boosts the estimations for the evidence preceding the categorical judgment resulting in increased sensitivity to that evidence we see in our data. Due to selective feedback from the accumulator circuit to early sensory regions encoding the evidence, the attractor state in accumulator networks may additionally modulate the processing of subsequent evidence in a selective fashion (15, 58) that depends on the consistency of that evidence with the initial choice, yielding a confirmation bias effect. These ideas should be explored with extended, hierarchical circuit models adapted to our task.

Another possibility, mutually nonexclusive with the above, is that transient neuromodulatory inputs to the decision circuits from brainstem arousal systems play a role in the overall sensitivity reduction to postdecisional evidence. The intermittent choices were always prompted after the first interval by the experimenter and under uncertainty. This is a setting in which the cortical decision circuits might not yet have reached a stable decision state on individual trials. In such a setting, the release of neuromodulators in the cortex induced by the choice may push decision circuits into an attractor state (1). Note that this effect is different from a gain modulation of sensory responses, which should increase evidence sensitivity, provided that the neuromodulatory input is still present when the new evidence arrives. Our pupil results, although exploratory and limited in nature, are roughly consistent with this idea: we found bigger pupil responses during the intermittent choice than the evidence-independent button press, and the individual amplitude of that choice-related response was negatively related to the individual sensitivity to subsequent evidence. Because of the task-related limitations of the current pupil analyses (outlined in results), the pupil results should be regarded as a starting point for future investigations into the role of phasic arousal in the weighting phenomena studied here.

Although our task design allows for assessing so far understudied aspects of decision-making, it also has limitations that open up alternative possibilities for explaining our findings. First, in all the No-Choice trials, the intermittent button press was followed by additional evidence in the second interval, whereas a proportion of Choice trials (50% in Perceptual task and 66% in Numerical task) ended with feedback about the categorical judgment without additional evidence. Consequently, participants may have expected a second stimulus on the No-Choice, but not the Choice trials, which may have affected (improved) the sensitivity for the upcoming stimulus (75–78). Second, and relatedly, participants may have only expected feedback on Choice trials, but not on No-Choice trials, again with differential effects on subsequent evidence processing (79, 80). Third, our task entailed a key role of certain forms of memory, as it required participants to store some format of representation of the evidence from the first interval in short-term memory across the intermittent response interval, in order to combine it with the second interval evidence. Indeed, evidence accumulation can be based not only on current sensory input but also on information from the visual processing pipeline (81, 82) or even stored in iconic memory for up to 500 ms (83), and working memory plays a critical role in related tasks (40, 84). In our task, the necessity to form and report a categorization judgment on Choice trials may have interacted with this memory in ways that contribute to the pattern of results. However, it is worth noting that neither of these alternate mechanisms can account for the confirmation bias observed in the task (42) without requiring additional assumptions. Future investigations should disambiguate between these different explanations by removing the feedback trials from the task design and verifying if participants exhibit the observed changes in sensitivity to the second stimulus interval.

To conclude, we have shown that intermittent choices on protracted streams of decision evidence have versatile and coupled effects on evidence accumulation, which lead to a reweighting of evidence sensitivity over time as well as to confirmation bias. These insights open the door for connecting laboratory studies of decision-making to realistic settings requiring protracted evaluation of time-varying evidence in multiple successive decisions.

## GRANTS

This research was supported by the German Academic Exchange Service (DAAD) (to A. E. Urai), a European Research Council Starting Grant under the European Union’s Horizon 2020 research and innovation program (Grant 802905) (to K. Tsetsos), and the following grants from the German Research Foundation (DFG): DO 1240/2-1, DO 1240/2-2, DO 1240/3-1, and DO 1240/4-1 (all to T. H. Donner). We acknowledge computing resources provided by Dutch Research Council (NWO) Physical Sciences.

## DISCLOSURES

No conflicts of interest, financial or otherwise, are declared by the authors.

## AUTHOR CONTRIBUTIONS

M.U. and T.H.D. conceived and designed research; A.E.U. performed experiments; B.C.T. and A.E.U. analyzed data; B.C.T., A.E.U., Z.Z.B., N.B., and K.T. interpreted results of experiments; B.C.T. prepared figures; B.C.T. and T.H.D. drafted manuscript; B.C.T., A.E.U., Z.Z.B., N.B., K.T., M.U., and T.H.D. edited and revised manuscript; B.C.T., A.E.U., Z.Z.B., N.B., K.T., M.U., and T.H.D. approved final version of manuscript; T.H.D. acquired funding for the research.

## ENDNOTE

At the request of the authors, readers are herein alerted to the fact that additional materials related to this manuscript may be found at https://doi.org/10.6084/m9.figshare.7048430, https://datadryad.org/resource/doi:10.5061/dryad.40f6v, https://doi.org/10.6084/m9.figshare.14039294, and https://github.com/BharathTalluri/choice-commitment-bias. These materials are not a part of this manuscript and have not undergone peer review by the American Physiological Society (APS). APS and the journal editors take no responsibility for these materials, for the website address, or for any links to or from it.
